# Developmental cognitive neuroscience using latent change score models: A tutorial and applications

**DOI:** 10.1016/j.dcn.2017.11.007

**Published:** 2017-11-22

**Authors:** Rogier A. Kievit, Andreas M. Brandmaier, Gabriel Ziegler, Anne-Laura van Harmelen, Susanne M.M. de Mooij, Michael Moutoussis, Ian M. Goodyer, Ed Bullmore, Peter B. Jones, Peter Fonagy, the NSPN Consortium, Ulman Lindenberger, Raymond J. Dolan

**Affiliations:** a Max Planck Centre for Computational Psychiatry and Ageing Research, London/Berlin; b MRC Cognition and Brain Sciences Unit University of Cambridge, Cambridge, 15 Chaucer Rd, Cambridge CB2 7EF; c Center for Lifespan Psychology, Max Planck Institute for Human Development, Berlin, Germany; d Institute of Cognitive Neurology and Dementia Research, Otto-von-Guericke-University Magdeburg, Germany; e German Center for Neurodegenerative Diseases (DZNE), Magdeburg, Germany; f Department of Psychiatry, University of Cambridge, United Kingdom; g Department of Psychological Methods, University of Amsterdam; h The Wellcome Centre for Human Neuroimaging, University College London, London WC1N 3BG, United Kingdom; i Cambridgeshire and Peterborough National Health Service Foundation Trust, Cambridge, CB21 5EF, United Kingdom; j ImmunoPsychiatry, GlaxoSmithKline Research and Development, Stevenage SG1 2NY, United Kingdom; k Medical Research Council/Wellcome Trust Behavioural and Clinical Neuroscience Institute, University of Cambridge; l Research Department of Clinical, Educational and Health Psychology, University College London; m See supplementary information for a full list of contributors; n European University Institute, San Domenico di Fiesole (FI), Italy

**Keywords:** Latent change scores, Longitudinal modelling, Development, Individual differences, Structural equation modelling, Adolescence

## Abstract

Assessing and analysing individual differences in change over time is of central scientific importance to developmental neuroscience. However, the literature is based largely on cross-sectional comparisons, which reflect a variety of influences and cannot directly represent change. We advocate using *latent change score* (LCS) models in longitudinal samples as a statistical framework to tease apart the complex processes underlying lifespan development in brain and behaviour using longitudinal data. LCS models provide a flexible framework that naturally accommodates key developmental questions as model parameters and can even be used, with some limitations, in cases with only two measurement occasions. We illustrate the use of LCS models with two empirical examples. In a lifespan cognitive training study (COGITO, N = 204 (N = 32 imaging) on two waves) we observe correlated change in brain and behaviour in the context of a high-intensity training intervention. In an adolescent development cohort (NSPN, N = 176, two waves) we find greater variability in cortical thinning in males than in females. To facilitate the adoption of LCS by the developmental community, we provide analysis code that can be adapted by other researchers and basic primers in two freely available SEM software packages (lavaan and Ωnyx).

## Highlights

• We describe Latent change score modelling as a flexible statistical tool.
• Key developmental questions can be readily formalized using LCS models.
• We provide accessible open source code and software examples to fit LCS models.
• White matter structural change is negatively correlated with processing speed gains.
• Frontal lobe thinning in adolescence is more variable in males than females.

## 1 Introduction

*When thinking about any repeated measures analysis it is best to ask first, what is your model for change?* (*McArdle, 2009, p. 579*)

Developmental cognitive neuroscience is concerned with how cognitive and neural processes change during development, and how they interact to give rise to a rich and rapidly fluctuating profile of cognitive, emotional and behavioural changes. Many, if not all, central questions in the field can be conceived as related to the temporal dynamics of multivariate brain-behaviour relations. Theories in developmental cognitive neuroscience often implicitly or explicitly suggest causal hypotheses about the direction of the association between variables of interest, the temporal precedence of their emergence, and the likely consequences of interventions. For instance, the *maturational viewpoint* (e.g. Gesell, 1929; cf. Johnson, 2011, Segalowitz and Rose-Krasnor, 1992) proposes that development of key brain regions (e.g. the frontal lobes) is a necessary precondition to acquiring psychological capacities (e.g. cognitive control or inhibition). This represents a clear causal pathway, where developmental change in neural regions precedes, and causes, changes in faculties associated with those regions (also known as *developmental epigenesis*). This is contrasted with *interactive specialisation theory* (Johnson, 2011), where *probabilistic epigenesis* posits bidirectional causal influences from mental function to brain structure and function. These competing theories make explicit claims about the temporal order of development, as well as the causal interactions between explanatory levels. Similarly, *developmental mismatch theory* (Ahmed et al., 2015, Mills et al., 2014, Steinberg, 2008, van den Bos and Eppinger, 2016) suggests that a key explanation of risk taking behaviour in adolescence is the delayed development of brain regions associated with cognitive control (e.g. the frontal lobe) compared to regions associated with mediating emotional responses (e.g. the amygdala). This too posits a clear brain-behaviour dynamic, where a mismatch between maturation in executive brain regions compared to emotion systems is hypothesized to affect the likelihood of certain (mal)adaptive behaviours. Empirical examples of such questions show, for instance, that frontoparietal structural connectivity (but not functional connectivity) determined longitudinal changes in reasoning ability (Wendelken et al., 2017).

An active area of research where cognitive or behavioural changes are presumed to precede changes in brain structure or function is that of *training-induced plasticity*. For instance, Bengtsson et al. (2005) found that degree and intensity of piano practice in childhood and adolescence correlated with regionally specific differences in white matter structure, and that this effect was more pronounced in developmental windows in which maturation was ongoing. This was interpreted as evidence of training-induced plasticity, suggesting that behavioural modifications (i.e. prolonged practice) preceded, and caused, measurable changes in white matter structure.^1^ More direct longitudinal evidence in 845 children scanned on two occasions suggests that grey matter volume and changes in white matter microstructure are slower in individuals with more severe psychiatric symptoms, but not vice versa. This is compatible with (although not conclusive evidence of) the hypothesis that differences in structural changes are consequences, not causes, of psychiatric symptoms (Muetzel et al., 2017).

Although such questions are characterized by a fundamental interest in temporal dynamics and causality, much of the literature is dominated by cross-sectional (age-heterogeneous) data that are ill equipped to resolve these questions (Lindenberger and Poetter, 1998, Lindenberger et al., 2011, Salthouse, 2014). For instance, individual differences in brain structure may precede differential changes in cognitive abilities (e.g. certain clinical conditions), or changes in cognitive abilities may trigger measurable changes in brain structure (e.g. learning-induced plasticity). Although these hypotheses imply radically different causal pathways and (potential) intervention strategies, they are often indistinguishable in cross-sectional data. Moreover, aggregated cross-sectional data can be affected by cohort effects (i.e. different populations) which in turn can lead to overestimates (e.g. cohort differences, Sliwinski et al., 2010), underestimates (e.g. selective attrition, training effects; Willis and Schaie, 1986), and even full reversals of the direction of effects observed between groups compared to within groups (Kievit et al., 2013). Most crucially, cross-sectional aggregations do capture change at the individual level, nor individual differences in intra-individual change (Baltes et al., 1977). Thus, they fail to directly address the most fundamental questions of developmental science: How and why do people differ in the way they develop?

The recent rapid increase in the study of large, longitudinal, imaging cohorts (Poldrack and Gorgolewski, 2014) provides unprecedented opportunities to study these key questions. Here we introduce a class of Structural Equation Models called *Latent Change Score Models* that are specifically tailored to overcome various weaknesses of more traditional approaches, and are well suited to address hypotheses about temporal, interactive dynamics over time.

## 2 Towards a model-based longitudinal developmental cognitive neuroscience

Structural equation modelling (SEM) combines the strengths of path modelling and latent variable modelling and has a long tradition in the social sciences (Bollen, 1989, Tomarken and Waller, 2005). Path modelling (an extension of (multiple) regression) allows for simultaneous estimation of multiple hypothesized relationships, specification of directed relations that correspond to hypothesised causal pathways, and models in which constructs may function as both dependent and independent variables. Latent variable modelling allows researchers to use observed (manifest) variables to make inferences and test theories about unobserved (latent) variables.

In offering a flexible framework for multivariate analyses SEM has several key strengths compared to other methods of analysis (Rodgers and Lee, 2010). First, SEM forces researchers to posit an explicit model, representing some hypothesized explanatory account of the data, which is then compared to the observed data (usually a covariance matrix, or a covariance matrix and a vector of means). The extent to which the hypothesized model can reproduce the observations is adduced as evidence in favour of, or against, some proposed model of the construct under investigation. Moreover, SEM helps make researchers aware of assumptions that may be hidden in other approaches (e.g., assumptions of equal variances across groups).^2^ Second, by using latent variables researchers can account for measurement error in observed scores. This strategy not only increases power to detect true effects (van der Sluis et al., 2010), but also offers greater validity and generalizability in research designs (Little et al., 1999). Specifically, it can be used to test for bias across subgroups (e.g. tests functioning differently for different subgroups, Wicherts et al., 2005) and biased estimates across developmental time (Wicherts et al., 2004), and improve the use of covariates (Westfall and Yarkoni, 2016).

In recent decades, various extensions of SEM have been developed for longitudinal, or repeated measures, data (McArdle, 2009). More traditional techniques (e.g. repeated measures ANOVA) are rarely tailored to the complex error structure of longitudinal data, neglect individual differences and are not developed explicitly to test the predictions that follow from causal hypotheses across a whole set of variables simultaneously. The longitudinal SEM framework, closely related to general linear mixed modelling (Bernal-Rusiel et al., 2013, Rovine and Molenaar, 2001), is so flexible that many common statistical procedures such as *t*-tests, regressions, and repeated measures (M)ANOVA can be considered special cases of longitudinal SEM models (Voelkle, 2007). Common procedures in developmental cognitive (neuro)science including cross-lagged panel models or simple regressions (on either raw or difference scores) can be considered special cases of LCSM’s, but without various benefits associated with SEM such as reduction of measurement error and incorporation of stable individual differences (Hamaker et al., 2015).

Examples of longitudinal SEM include latent growth curve models, latent change score models, growth mixture models, latent class growth curve modelling and continuous time modelling (Driver et al., 2016, McArdle, 2009). In the next section we describe a specific subtype of longitudinal SEM known as the *Latent Change Score Models (LCS*, sometimes also called *Latent Difference Score* models) (McArdle and Hamagami, 2001b, McArdle and Nesselroade, 1994). This particular class of models is especially versatile and useful for researchers in developmental cognitive neuroscience as it can model change at the construct level, can be used with a relatively modest number of time points (a minimum of 2, although more are desirable) and is especially powerful for testing cross-domain (i.e. brain behaviour) couplings.

## 3 The Latent Change Score model

Latent Change Score models (McArdle and Hamagami, 2001b, McArdle and Nesselroade, 1994) are a powerful and flexible class of Structural Equation Models that offer ways to test a wide range of developmental hypotheses with relative ease. LCSMs have been used to considerable effect in developmental (cognitive) psychology to show a range of effects including that vocabulary affects reading comprehension but not vice versa (Quinn et al., 2015), that people with dyslexia show fewer intellectual benefits from reading than controls (Ferrer et al., 2010), that positive transfer of cognitive training generalizes beyond the item-level to cognitive ability (Schmiedek et al., 2010), that volume changes of the hippocampus and prefrontal white matter are reliably correlated in adulthood and old age (Raz et al., 2005), that an age-related decline in white matter changes is associated with declines in fluid intelligence (Ritchie et al., 2015) and that basic cognitive abilities such as reasoning and vocabulary show mutualistic benefits over time that may partially explain positive correlations among cognitive abilities (Kievit et al., 2017). One of the first applications of LCS in cognitive neuroscience showed that ventricle size in an elderly population predicted rate of decline on memory tests across a seven year interval (McArdle et al., 2004). There are several excellent tutorials on longitudinal SEM (Ghisletta and McArdle, 2012, Grimm, 2007, Jajodia and Archana, 2012, McArdle and Grimm, 2010, Petscher et al., 2016, Snitz et al., 2015, Usami et al., 2016, Zhang et al., 2015), and the approach we outline below builds heavily upon this previous work, where we illustrate LCS models specifically in the context of Developmental Cognitive Neuroscience. We will start with the simplest model, using one variable measured on two occasions, and then present four extensions of the model. These extensions will sequentially incorporate latent variables, add multiple coupled domains (cognitive and neural measures), extend to multiple time waves (latent growth- and dual change score models) and finally test for differences in multiple groups. After discussing each of the basic models in turn, we will cover methodological challenges including estimation, model fit, interpretation and model comparison. For each of the five types of models we discussed below, we provide example syntax that simulates data under a selected parameterisation and fits the model in question to the data. These scripts are freely available at the Open Science Framework https://osf.io/4bpmq/files/ to be used, modified and extended by the wider community.

### 3.1 Univariate latent change score model

Imagine a researcher studying a psychological variable of interest, repeatedly measured at two time points (T1 and T2) in a population of interest. A traditional way to examine whether scores of a group of individuals increased or decreased between T1 and T2 is performed by means of a paired *t*-test. Using some simple modifications, the LCS allows us to go beyond this traditional analysis framework even in this simplest case. The basic steps of a univariate latent change score model are as follows. To facilitate understanding, in the examples below, we will use informative notation (e.g. ‘COG’ for cognitive measures and ‘NEU’ for neural measures). For a more standard mathematical notation of the LCS we refer the reader to texts such as (McArdle, 2008, McArdle and Hamagami, 2001a, Newsom, 2015, Petscher et al., 2016). First, we conceptualize the scores of an individual *i* on the construct of interest *COG* at time *t* as being a function of an autoregressive component and some residual. By fixing the regression weight of *COGT2* on *COGT1* to 1, the autoregressive equation simplifies to

(1) $${COG}_{i,t2}\;={COG}_{i,t1\;}\;+{\Delta COG}_{i,1}\;$$

From this it follows that the change score is simply:

(2) Δ*COG_i,1_* = *COG*_*i,t*2_ − *COG*_*i,t*1_

The powerful step in the context of SEM is to define a latent change score factor *ΔCOG1*, which is measured by time point 2 with a factor loading fixed to 1. Doing so creates a latent factor that captures the change between time 1 and time 2. Finally, we can add an regression parameter *β* to the change score, which allows us to investigate whether the degree of change depends on the scores at time 1 as follows:

(3) Δ*COG_i_*_,1_ = *β*·*COG*_*i,t*1_

With this model in place we can address three fundamental questions. The first and simplest question is whether there is a reliable average change from T1 to T2. This is captured by the mean of the latent change factor, *μΔCOG1*. Under relatively simple assumptions this test is equivalent to a paired *t*-test (Coman et al., 2013). However, even this simplest implementation of the latent change score model yields two additional parameters of considerable interest. First, we can now estimate the *variance* in the change factor, *σ^2^ΔCOG1*, which captures the extent to which individuals *differ* in the change they manifest over time. Second, we can specify either a covariance or an autoregressive parameter *β* which captures the extent to which change is dependent, or *proportional*, to the scores at time one (this parameter can also be specified as a covariance if so desired). Note that if a regression parameter is included, the mean change should be interpreted conditional on the regression path – A covariance will yield the mean ‘raw’ change.

**Fig. 1:**
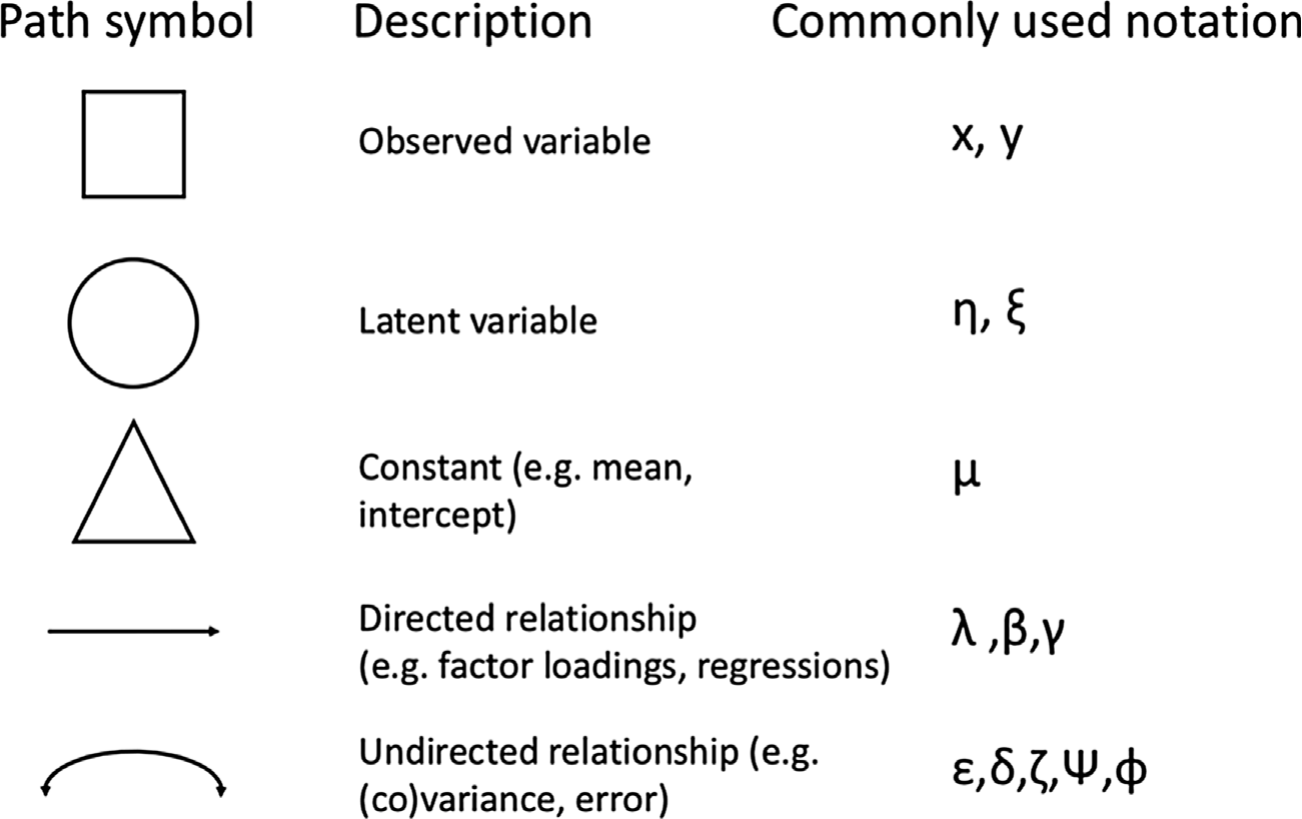
Basic path model notation.

**Fig. 2:**
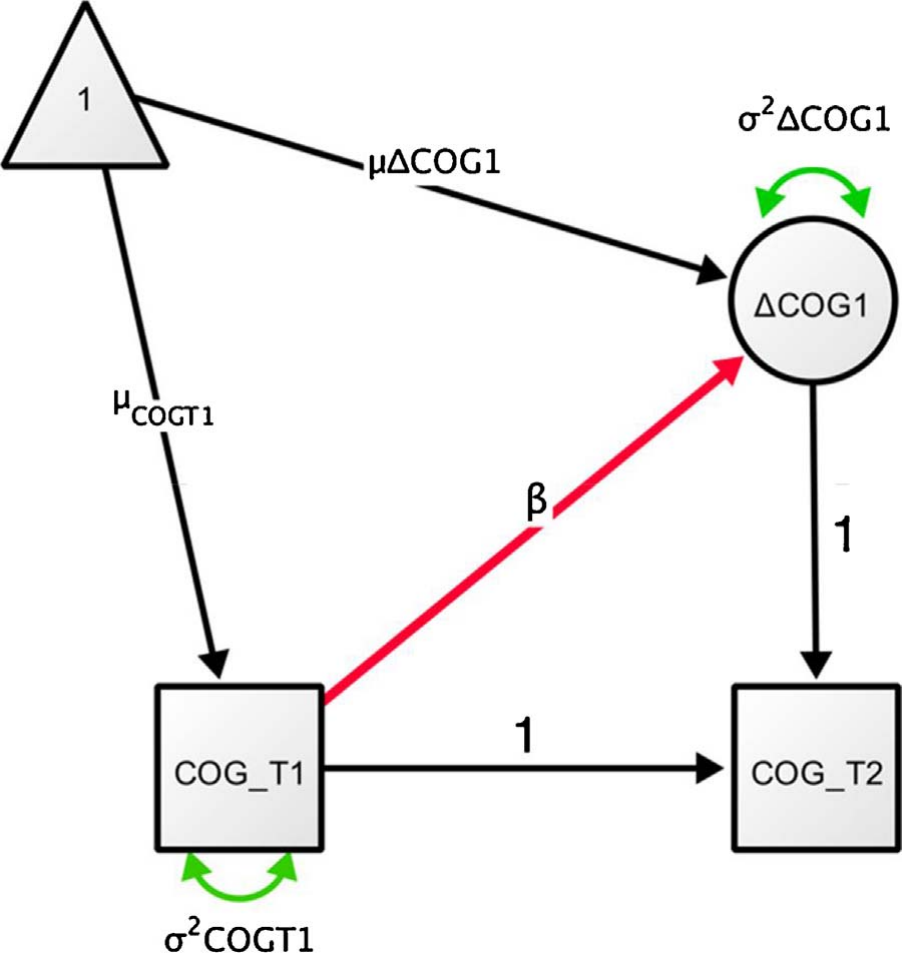
Univariate Latent Change Score Model. Variable COG is measured at two time points (COG_T1 and COG_T2). Change (**Δ**COG1) between the two timepoints is modelled as latent variable.

SEMs are often illustrated using *path models*. Such representations go back to Wright (1920), and allow researchers to represent complex causal patterns using simple visual representations. Fig. 1 shows the commonly employed symbols, meaning and notation. The simplest representation of the univariate latent change model is shown in Fig. 2, and can be fit to data measured on two occasions. As the autoregressive parameter between *COGT2* and *COGT1* is fixed to unity, we implicitly assume that the intervals are equidistant across individuals. Deviations from this assumption can be dealt with by rescaling scores (Ferrer and McArdle, 2004, p. 941) or, ideally, by using definition variables (Mehta and West, 2000) or continuous-time modelling approaches (Driver et al., 2016), which yield parameters that more easily generalize across different longitudinal designs. The model shown in Fig. 2 is *just identified*, that is, there are as many unique pieces of information that enter the model (two variances, two means and a covariance) as parameters to be estimated (one observed variance, one latent variance, one observed mean score, one latent mean score and one regression parameter). This means that although we can estimate this model, we cannot interpret model fit in isolation unless additional data (multiple waves, multiple domains or multiple indicators) are included. However, we can make use of parameter constraints, namely fixing certain parameters to zero and employ likelihood ratio testing of hypotheses about specific parameters. For instance, one can separately fit two similar models: once a model with the latent change variance parameter freely estimated, and once with the variance constrained to 0 (implying no differences in change). The difference in model fit will be chi-square distributed with *k* degrees of freedom, where *k* is the number of parameters constrained to equality (Neale, 2000; but see Stoel et al., 2006). If fixing the variance of change parameter to 0 leads to a significant drop in model fit, it would suggest that individuals change heterogeneously. However, it should be noted that this inference is only valid compared to the simpler model – a more extensive model with additional variables or time points may lead to different conclusions about heterogeneity in change. A more practical concern is that constraining variances parameters may lead to failure of model convergence which renders interpretation challenging – See Section 4.2 for more guidance. Similar procedures can be employed for any other parameter of interest or combinations of any number of parameters. The likelihood ratio test is especially suitable for parameters such as variances, as the simplifying assumptions of parameter significance tests such as the often-used Wald test may not hold (i.e. a variance cannot be negative). Next, we examine how to extend the LCS model to include latent variables.

### 3.2 Multiple indicator univariate latent change score model

The above example uses a single observed variable, which was assumed to be measured without error. We can easily extend this model to have an explicit measurement model by replacing the observed score with a *latent variable*, measured by a set of observed variables. We refer to this representation as a multiple indicator latent change score model, as our aim is to model change in the latent score rather than observed scores. To do so, we model a latent variable in the manner of a traditional confirmatory factor analysis, by expressing the strength of the association between the latent variable *COG* in individuals *i* (*i *= 1*,…N*) measured at times *t* (*t *= 1,…*t*) and the observed scores X (*j *= 1,…*j*) with factor loadings *λ* and error terms *δ* as follows:

(4) *X_ijt_* = *λ_jt_COG_it_* + *δ_ijt_*

**Fig. 3:**
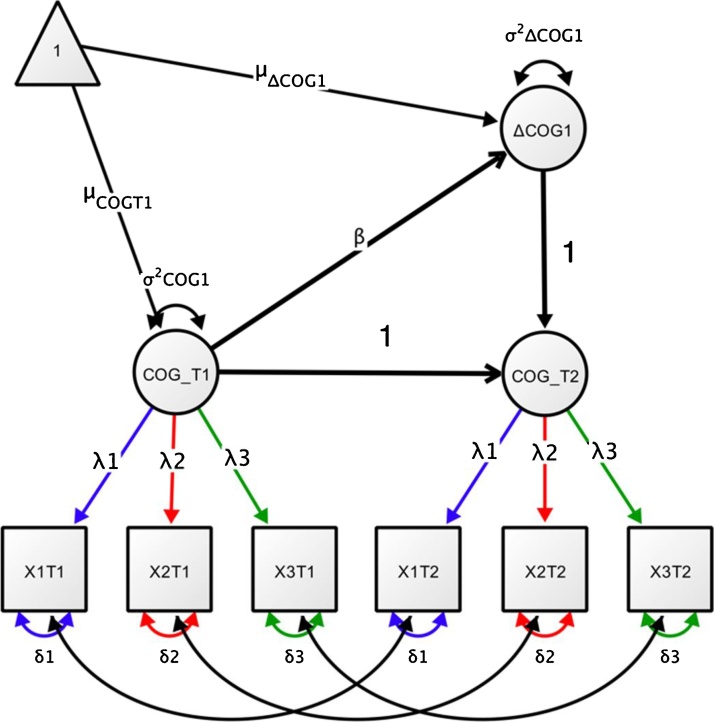
Multiple indicator univariate latent change score model. The latent construct of interest (COG) is measures at two time points (COG_T1 and COG_T2) each measured using three indicators (X1, X2, X3). We assume measurement invariance and correlated residual errors over time. See text for a detailed description of the model parameters.

A simple multiple indicator latent change score model is shown in Fig. 3. We model the mean, variance and autoregressive changes in *COG* as before, but now add a set of three (*X1-X3*) observed measurements on two occasions that each reflect the underlying cognitive construct of interest. Additionally, we allow for residual covariance of error terms across time points for each observed score with itself, represented as double-headed arrows. These so-called ‘correlated errors’ allow for indicator-specific variance across occasions and are generally included as default (Newsom, 2015, p. 103; Wheaton et al., 1977). This model is similar to the univariate latent change score model in terms of the key questions it can address (rate of change *μΔCOG1*, variance in change *σ^2^ΔCOG1*, and the relation between *COG1* and *ΔCOG1* captured by *β*), but includes the benefits of removing measurement error and establishing measurement invariance over time and (if necessary) across groups, improving inferences.

### 3.3 Bivariate latent change score model

**Fig. 4:**
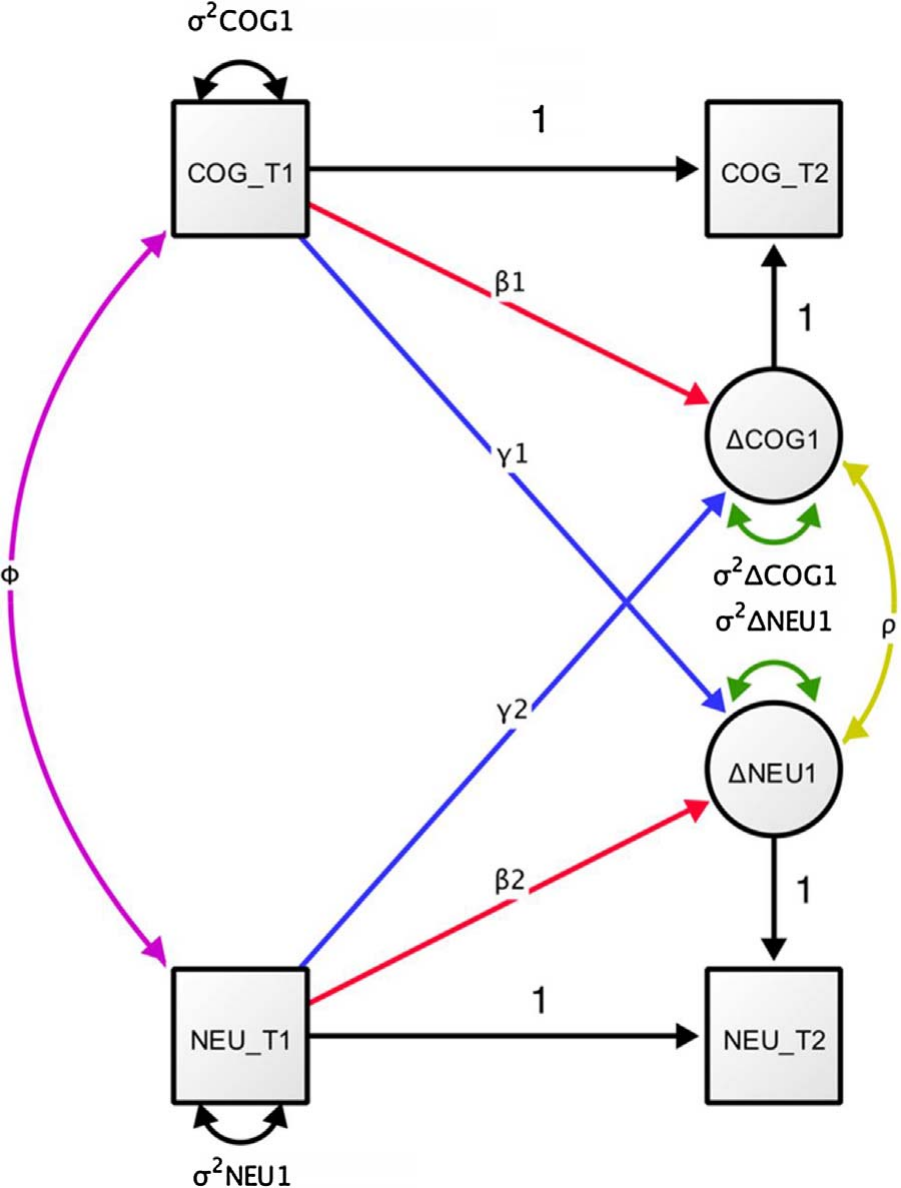
Bivariate Latent Change Score Model. Note: means are omitted for visual clarity.

A further extension of the latent change score model is to include a second (or third, fourth, etc.) domain of interest. For convenience in notation and graphical representation we will revert back to using only observed scores, but all extensions can and – where possible should – be modelled using latent (multiple indicator) factors. We can assume the second domain is some neural measure of interest (e.g. grey matter volume in a region of interest), measured on the same number of occasions as the cognitive variable (or variables). This allows for the investigation of a powerful concept known as *cross-domain coupling* (Fig. 4), that captures the extent to which *change* in one domain (e.g. *ΔCOG*) is a function of the starting level in the other (i.e. *NEUT1*). For instance, we can quantify the extent to which *cognitive changes* between T1 and T2 are a function of brain structure (*γ*2) and cognition (*β*1) at T1 as follows:

(5) *ΔCOG1 = β*1·*COG_i,t1_ + γ*2·*NEU*_*i,t*1_

The implications for testing theories in developmental cognitive neuroscience should be immediately clear: the dynamic parameters, shown in red and blue in Fig. 4, capture the extent to which changes in cognition are a function of initial condition of brain measures, vice versa or both. Likelihood ratio tests or Wald tests of these dynamic parameters (brain measures affecting rates of change in cognition, or cognitive abilities affecting neural changes) furnish evidence for, or against, models that represent uni- or bidirectional hypothesized causal influences. As is clear from Fig. 4, the bivariate latent change score model can capture at least four different brain-behaviour relations of interest. First, we have brain-behaviour covariance at baseline (shown in purple), the main focus in traditional (developmental) cognitive neuroscience. Second, we have cognition to brain coupling (shown in blue, labelled *γ1*), where T1 scores in cognition predict the rate, or degree, of change in brain structure. For instance, the degree of childhood piano practice affected white matter structure (*ΔNEU1*) would predict a substantial cognition-to-neural coupling parameter *γ1* (Bengtsson et al., 2005). Third, we have brain structure predicting rate, or degree, of cognitive change (shown in blue, labelled *γ2*). For example, McArdle et al. (2004) showed that ventricle size in an older population predicted rate of memory decline across an interval of 7 years. Finally, we have an estimate of correlated change (shown in yellow), reflecting the degree to which brain and behaviour changes co-occur after taking into account the coupling pathways. For instance, Gorbach et al. (2016) observed correlated change between hippocampal atrophy and episodic memory decline in older adults. More generally, correlated change may reflect a third, underlying variable influencing both domains. The bivariate latent change score provides a powerful analytic framework for testing a wide range of hypotheses in developmental cognitive neuroscience in a principled and rigorous manner.

### 3.4 Bivariate dual change score model

So far, we focused on the simplest instance of longitudinal data, namely where data is measured on two occasions. This is likely to be, for the foreseeable future, the most common form of longitudinal dataset available to researchers in developmental cognitive neuroscience, and yields many benefits compared to both cross-sectional data analyses and more traditional techniques such as cross-lagged panel models or change score regression (see Section 4 for more detail). However, with a greater number of timepoints, extensions within the framework of LCS models makes it easy to capture more fine-grained dynamic processes within and across domains. For instance, a sufficient number of timepoints allows one to fit what is known as a *dual change score model* (Ghisletta and Lindenberger, 2003). In this model, we specify an additional latent variable, S (for slope), that captures the global increase or decrease across all time points. This latent variable is measured by the successive change scores *ΔCOGt*, by specifying factor loadings (α) to capture a range of dynamic shapes such as linear increase or decrease. The factor loadings of the slope factor on the constant change parameter can be fixed to a priori values to capture a range of growth processes including linear (all 1) or accelerating change (e.g. 1,2,3) – however, due to identification constraints, they cannot generally be freely estimated from the data.

**Fig. 5:**
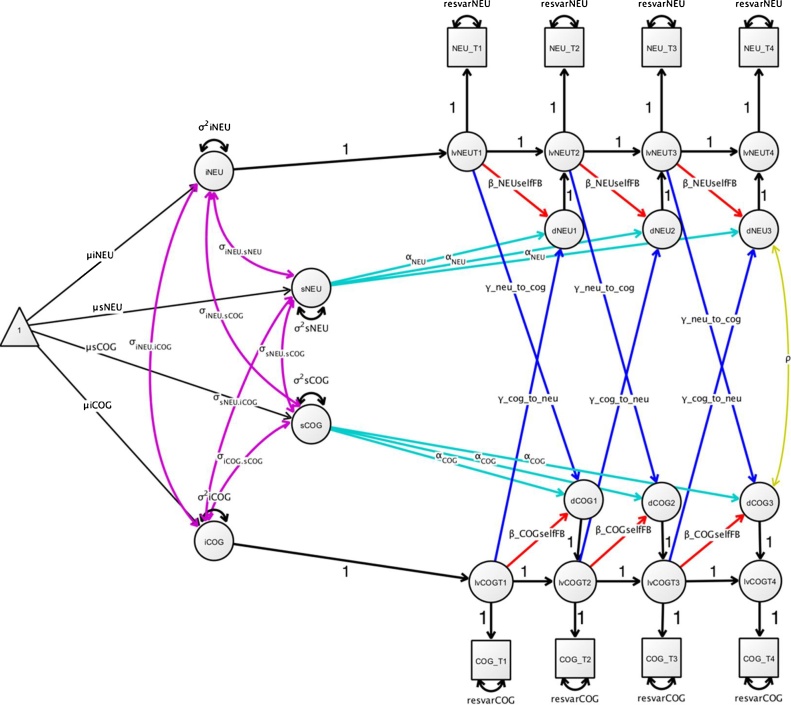
Bivariate Dual Change Score Model. This more complex latent change score model captures both the stable change over time in the form of slopes (sCOG and sNEU), as well as more fine-grained residual changes. Note this model incorporates latent variables at each timepoint – See Newsom (2015, p. 135) for more detail.

The ‘dual’ aspect of this model enters by separating the global process of change captured by the slope from the more local, time point-to-time point deviations from this trajectory denoted by the self-feedback (*β*, red pathways in Fig. 5) and cross-domain coupling (*γ*, blue pathways in Fig. 5) parameters. When modelled together with a neural variable the bivariate dual change score, shown graphically in Fig. 5, can be expressed as follows

(6) Δ*COG_i,t_ = α_COG_*·*sCOG_i_ *+* β*1·*COG_i,t_ + γ*2·*NEU_i,t_*

This (bivariate) dual change score model is a general approach that can capture both general trends and more fine-grained temporal dynamics. This can be especially useful when trying to separate a known, more stable change occurring during development (e.g. global cortical thinning) from more high-frequency fluctuations. The dual change score model has been used in a behaviour-only context to show (a) that vocabulary influences changes in reading ability (but not vice versa) (Quinn et al., 2015); (b) bivariate dynamic coupling between subjective and objective memory problems in an ageing population (Snitz et al., 2015); (c) within-person trial-to-trial RT variability predicts cognitive decline in old and very old age (Lövdén et al., 2007); and (d) perceptual speed decline in old age predicts decline in crystallized intelligence to a greater extent than vice versa (Ghisletta and Lindenberger, 2003).

### 3.5 Multigroup latent change score models: manifest groups, mixtures and intervention studies

The LCSM provides a comprehensive framework to model both within-person change across time and between-person variability in change. A final, powerful extension that can be applied to any latent change score (or SEM) model is the possibility for *multigroup* comparisons. Parameter estimates of a LCSM are valid under the assumption that there is no model misspecification and the sample is drawn from a single, homogeneous population. In practice, however, our samples may be a mixture of participants from different populations (e.g., children and older adults; men and women, low vs. high SES). There are several ways to address sample heterogeneity depending on the assumptions we are willing to make and the strength of our theoretical reasoning concerning sample heterogeneity. First and foremost, approaches to model heterogeneity can be classified by whether heterogeneity is assumed to be observed or unobserved.

When heterogeneity is observed in a confirmatory modelling approach, hypothesis testing is concerned with finding statistical evidence for difference in the key parameters of the LCSM. Cross-sectional analyses often use traditional methods such as (M)ANOVA’s to focus on simple parameters of interest, such as the mean scores on some outcome of interest. In a SEM context, it is relatively easy to compare *any* parameter of interest in a (dynamic) model across groups. To do so, one simply imposes equality constraints on the parameter of interest and compares the model where the parameter of interest is freely estimated to a constrained model as described above. Relatively sophisticated questions about changing relations between constructs, developmental dynamics and group differences can be addressed with this simple yet powerful test, with previous investigations comparing regression coefficients (e.g. the negative effect of relational bullying on friendships is stronger in boys than in girls, van Harmelen et al., 2016), or dynamic growth components (e.g. boys and girls show differential response dynamics following divorce, Malone et al., 2004). In cases where a large number of covariates are potentially relevant but we have no strong theories to guide us, more exploratory techniques such as SEM trees (Brandmaier et al., 2013) and SEM forests (Brandmaier et al., 2016) allows researchers to hierarchically split empirical data into homogeneous groups sharing similar data patterns, by recursively selecting optimal predictors of these differences from a potentially large set of candidates. The resulting tree structure reflects a set of subgroups with distinct model parameters, where the groups are derived in a data-driven way. Group divisions are often based on observed variables, but if heterogeneity is assumed to be unobserved, researchers may turn to *latent mixture models* (McLachlan and Peel, 2005, but for a cautionary note see Bauer, 2007).

An often overlooked application of LCS and SEM models is in intervention studies (McArdle, 1994). We can treat grouping of participants into treatment and control groups in precisely the same way as traditional grouping variables such as gender or education, and compare all model parameter using likelihood ratio tests. For instance, Raz et al. (2013) showed less cerebellar shrinkage in a cognitive training intervention group than in controls, and Maass et al. (2015) using SEM to demonstrate correlated change in between fitness improvement and memory. By modelling time by group interaction in a SEM context, one can use multiple indicator latent change score models to derive error-free effect sizes of the treatment effect, by subtracting average latent change in control group from average latent change in the treatment group for latent constructs (e.g. Schmiedek et al., 2010, Schmiedek et al., 2014). Once researchers have decided on which model best matches their developmental hypothesis and is compatible with the available data, it is time to estimate and interpret the model.

## 4 Challenges and limitations

### 4.1 Model fit, model estimation and model comparison

Once a model has been specified for a suitable dataset, a researcher will estimate the free parameters in the model. The most common approach to parameter estimation in SEM is maximum likelihood, under the assumption of multivariate normality. The extent to which this assumption is violated can bias results, although adjusted model fit indices have been developed to account for deviations from (multivariate) normality (e.g. Satorra-Bentler or Yuan-Bentler-scaled test statistics; Rosseel, 2012). Note that these methods only adjust fit indices, not standard errors, which may also be affected by deviations from (multivariate) normality – Various additional methods such as Huber-White standard errors can be used to address this challenge and are implemented in almost all SEM packages (including lavaan, see Rosseel (2012, p. 27) for more detail). Alternatively, other estimation strategies can be used to estimate non-continuous or non-normal outcomes (e.g., threshold models or weighted-least-squares estimators for ordinal data) but as a detailed investigation of this issue is beyond the scope of this tutorial we refer the reader to additional resources (Kline, 2011, Olsson et al., 2000, Rosseel, 2012, Schermelleh-Engel et al., 2003).

A key intermediate step in longitudinal SEM in the case of measurement models (e.g. Fig. 3) is to provide evidence for *measurement invariance*, that is to ensure that the same latent construct (e.g. general intelligence) is measured in the same manner across time or across groups (Meredith, 1993, Millsap, 2011, Vandenberg and Lance, 2000, Wicherts et al., 2004). In other words, we want the relationship between levels of the latent variables and the observed scores to be equal across time, even when latent scores themselves are increasing or decreasing on average. Failing to establish measurement invariance can lead to incorrect conclusions about latent variables, their growth over time, and their relations to other variables (Ferrer et al., 2008). In longitudinal SEM, a series of increasingly strict tests (Widaman et al., 2010) can be applied to ensure measurement invariance. Conventionally, this is done by establishing equality constraints over time, by sequentially equating the factor loadings (*λ*_*jt*1_ = *λ*_*jt*2_), error terms (*δ*_*jt*1_ = *δ*_*jt*2_) and intercepts across time or groups. Such constraints can be shown graphically in model representations – For instance in Fig. 3, these equality constraints are shown by designating the same factor loading with a single label (e.g. λ1) or colour across loading (e.g. blue) across time points. Recent work shows that the CFI (comparative fit index, see for more detail below) is a practical way to test for measurement invariance across increasingly strict models created by imposing a specific sequence of model constraints (Cheung and Rensvold, 2002). If measurement invariance is violated, the extent to which inferences are affected and possible remedies using ‘partial measurement invariance’ are discussed in (Vandenberg and Lance, 2000).

A key strength of estimation in SEM is the treatment of missing data (Enders, 2001). Assuming data is either Missing Completely At Random (MCAR) or Missing At Random (MAR), which means the missing data can only be dependent on variables also measured within the same dataset (e.g. if differences in dropout are gender specific, and gender is assessed), Full Information Maximum Likelihood (FIML) can be used to estimate a model on the full dataset (including subjects with incomplete data) (Baraldi and Enders, 2010, Enders, 2001, Wothke, 2000). Using FIML for missing data (under multivariate normality) maximizes the utility of all existing data, decreases bias and increases statistical power compared to (for instance) omitting incomplete cases (‘complete case analysis’; Baraldi and Enders, 2010). In direct comparisons, FIML usually performs as well or better than alternative methods such as multiple imputation (MI) (Larsen, 2011, von Hippel, 2016). A practical benefit of FIML compared to MI is the stability of estimation across uses, whereas multiple imputation depends on stochastic sampling and will yield a (slightly) different estimate every time. Moreover, combining information across different imputations can be challenging, although this has been automated for SEM with lavaan via the auxiliary package ‘semTools’ (Jorgensen et al., 2015).

Once model estimation has finished (which may take anywhere from fractions of a second to days), a wide range of model fit indices are available to assess model fit (Kline, 2011, Schermelleh-Engel et al., 2003). Generally, these metrics quantify the deviation between the observed and implied covariance matrix. Model fit metrics include a simple test of deviation from perfect model fit (the chi square test), indices that compare the degree to which the proposed model better fits the data (e.g. the CFI and TLI) compared to some baseline model (which typically is a model in which there are no correlations between measurements; and good fit represents the degree to which covariation in the empirical data is reliably modelled), and measures that quantify some standardized measures of the deviation between the observed and implied covariance matrices (e.g. SRMR or the RMSEA). Fit indices can be affected by a range of model and data properties including sample size, measurement quality, estimation method, misspecification and more (Fan et al., 1999, McNeish et al., 2017, Moshagen and Erdfelder, 2016). Competing models can be compared using traditional likelihood ratio test if models are nested (Neale, 2000), or specialized version of the LRT for non-nested models (Merkle et al., 2016). Other approaches to model comparison include the use of model fit indices such as CFI and RMSEA (Usami et al., 2016), or information based metrics such as the AIC and BIC (Aho et al., 2014, Wagenmakers and Farrell, 2004). A relatively new question inspired by cognitive neuroscience will be how to best conduct model selection and model comparison in procedures such as voxelwise modelling from brain image data (Madhyastha et al., n.d.) which may require hundreds or thousands of model comparisons or extended measurement models of spatially correlated observations – This at present remains an open challenge.

Debates concerning the optimal way to assess and interpret model fit, which thresholds to use or how to best compare models are wide ranging and beyond the scope of this tutorial, for further details see (Barrett, 2007, Fan et al., 1999, Hayduk et al., 2007, Schermelleh-Engel et al., 2003, Steiger, 2007). Common advice includes reporting multiple (types of) fit indices to allow for a more holistic assessment such as reporting raw χ^2^, CFI and RMSEA (Schermelleh-Engel et al., 2003). Recommended sources for a wide range of (longitudinal) SEM topics include McArdle (2009), Newsom (2015), Hoyle (2014), Little (2013), Voelkle and Oud (2015), Driver et al. (2016) and Voelkle (2007), as well as the tutorials cited above. Other useful resources are SEM-oriented email groups such as SEMNET (http://www2.gsu.edu/∼mkteer/semnet.html) or package focused help groups (e.g. https://groups.google.com/forum/#!forum/lavaan).

### 4.2 Convergence and improper solutions

Although SEM in general and LCS in particular cover a broad and flexible range of models and techniques, these techniques have various limitations. Below we outline a subset of commonly faced challenges. After specifying an LCS model, researchers will use a particular estimation procedure, typically Maximum Likelihood, to provide estimates for the parameters in the model. However, in contrast to simpler methods such as *t*-tests and simple regressions, model estimation may fail to converge. Common causes of a failure to converge include small sample sizes, overly complex models, poor starting values, inappropriate estimators, large amounts of missing data, data input errors, or misspecified models (Fan et al., 1999, Jackson, 2007, Wothke, 1993). A particular challenge in the context of latent change score models (and closely related models such as linear mixed models) is that of estimating or constraining variances terms (close) to 0 (for instance, constraining the variance to 0 in the simplest univariate latent change score model will generally lead to non-convergence even if the change scores between T1 and T2 are identical across individuals). Classical estimation methods such as Maximum Likelihood have relatively high non-convergence rates in such scenarios, and non-convergence has often (erroneously) been interpreted as evidence that the model is necessarily inappropriate. Moreover, the likelihood ratio test may not behave appropriately in scenarios including such ‘boundary values’ (Stoel et al., 2006). One promising solution is Bayesian estimation, which has been suggested to have considerably less estimation problems (Eager and Roy, 2017, Merkle and Rosseel, 2015, Muthén and Asparouhov, 2012, Van Erp et al., 2017). Secondly, even if estimation does converge, model fit may be ‘improper’ in various ways. Such improper solutions may include negative variances, standardized estimates that (far) exceed 1 (sometimes referred to as ‘Heywood cases’, but see Jöreskog, 1999), or matrices that are ‘non-positive definite’ (Wothke, 1993). No unique cause underlies these problems, nor does a single solution exist that applies in all cases, but various remedies may help. These including providing plausible starting values for parameters to aid estimation, increasing sample sizes, using a different estimator, or constraining parameters to 0 or to (in)equality where appropriate – for example, variances which are estimated just below zero might be constrained to zero so that they remain within appropriate bounds. For further reading on challenges and solutions of model convergence and inappropriate solutions we recommend (Eager and Roy, 2017, Fan et al., 1999, Gerbing and Anderson, 1987, Wothke, 1993).

### 4.3 Power and sample size

A challenge closely related to model fit and model comparison is that of statistical power and the associated question of sample size (Cohen, 1988). One often encounters rules of thumb (such as “one should have 20 subjects per SEM parameter”), which typically are misleading and never capture the full story. Here, we advise against such heuristics. When designing a longitudinal study, there are many more design decisions that directly affect statistical power, such as indicator reliability, true effect size, or the number and spacing of measurement occasions (Brandmaier et al., 2015), and strategies exist to improve power without necessarily increasing sample size (Hansen and Collins, 1994). Longitudinal models have successfully been fit to as few as 22 subjects (Curran et al., 2010), but as for all statistical approaches, larger sample sizes will generally lead to more robust inferences. Although factors determining statistical power in latent growth models are reasonably well understood (Hertzog et al., 2006, Hertzog et al., 2008, Oertzen et al., 2010, Rast and Hofer, 2014, von Oertzen and Brandmaier, 2013, von Oertzen et al., 2015), we know of no empirical simulation studies that may serve as guidelines for the power of (bivariate) LCSM. Currently, statistical power for a specific LCSM design (including a hypothesized true effect and sample size) can be approximated mathematically (Satorra and Saris, 1985) or by computer-intensive simulation (Muthén and Muthén, 2002). For a more general approach to the question of model selection, model comparison and parameter recovery we have included a flexible, general script that can allow users to generate a dataset under known conditions from a given model, and fit one or more models to this dataset^3^. This simple approach should allow anyone to examine compare parameter recovery, convergence rates, statistical power, model selection and model fit under a range of effect sizes, sample sizes and missingness to facilitate appropriate study planning.

### 4.4 Inference and causality

Once model convergence and adequate model fit are obtained, the most daunting step is that of (causal) inference: What may and may not be concluded based on the results? One goal of SEM is to test predictions derived from causal hypotheses about the process that generated the data, represented as a model. That is, although SEM (nor any other correlation-based technique) cannot directly demonstrate causality or causal processes, it can be used as a statistical tool for deriving model-based predictions of causal hypotheses, and examine the extent to which the data disconfirms these hypotheses (Bollen and Diamantopoulos, 2015, Pearl, 2000). However, inferring causality is, unsurprisingly, non-trivial. A first and most fundamental challenge, not specific to SEM, is that of *model equivalence*, known in philosophy of science as the ‘the underdetermination of theory by data’ (e.g. Newton-Smith and Lukes, 1978). In the context of SEM, it has been shown formally that any observed data pattern is compatible with many different data generating mechanisms (Raykov and Penev, 1999). In other words, even if a model fits well in a sufficiently large dataset, that in and of itself is not conclusive evidence for the (causal) hypotheses posited by the model. Moreover, in a longitudinal context, modelling choices and omitted variables can affect, and even spuriously invert, causal direction and temporal ordering (Usami et al., 2016) as well as the magnitude (Voelkle and Oud, 2015) of effects. Although recent years have seen a consistent trend away from causal language with recommendations to move away from the term ‘causal modelling’ as shorthand for SEM (Kline, 2011), for a spirited defence as well as a historically informed overview of the causal history and foundations of SEM, see Pearl (2012). The most commonly accepted solution is that model inferences, including causality, should come from a convergence of robust empirical evidence guided by theoretical motivations, and ideally be validated by interventions whenever possible.

Finally, although SEM is commonly used as a technique to test whether data is (provisionally) compatible with a particular (causal) hypothesis, in practice SEM spans a range of approaches from almost entirely exploratory to confirmatory. In the context of LCS models, non-trivial misfit may be amended by model re-specification to achieve acceptable fit. One approach to improve model fit is the examination of ‘modification indices’ – The expected improvement in model fit if a currently constrained parameter was freely estimated. As this is generally purely data-driven, this practice may adversely affect interpretability and generalization to independent datasets, so should be exercised with caution (MacCallum et al., 1992). Other approaches include the addition of cross-loadings, the elimination of non-significant structural paths, constraining or equating parameters or the exclusion of poorly performing measurement indicators. All of those changes may be defensible, but researchers should be explicit about any modifications that were made purely to improve model fit, so as to be able to assess the evidence in favour of the ‘final’ model appropriately (Bentler, 2007, MacCallum et al., 1992).

### 4.5 LCS versus alternative models

Structural equation modelling is an extremely general framework to study differences and change, and shares foundations with other analytic approaches. Previous work has shown similarities and even equivalences with other analytical traditions such as multilevel- and linear mixed modelling (Bauer, 2003, Curran, 2003, Rovine and Molenaar, 2001). However, even when models are mathematically equivalent in principle, they may still diverge in practical terms, such as ease of model specification and common defaults – McNeish and Matta (2017) examine in which situations linear mixed models versus SEM approaches are a better choice. An overarching treatment by Voelkle (Voelkle, 2007) has shown how a wide variety of analytical techniques ranging from *t*-tests to MANOVAs can be (re)written as special cases of the latent growth curve model (which is itself a special case of the latent change score model). For instance, (Coman et al., 2013) shows how a basic LCS is a special case of the paired *t*-test. Similarly, simple forms of the bivariate latent change score model can be rewritten as a special case of a cross-lagged panel model, namely the recently proposed random-intercept cross-lagged panel model (Hamaker et al., 2015), and the autoregressive cross-lagged factor model is equivalent to a latent change score model when slope factor scores are equivalent across individuals (Usami et al., 2016).

Grimm (2007) used three popular longitudinal models, the bivariate latent growth curve model, the latent growth curve with a time-varying covariate, and the bivariate dual change score growth model, to examine the same dataset concerning the relation between depression and academic achievement. Although the three models yielded different results, Grimm (2007) illustrates how each of the three approaches answer slightly different developmental questions, illustrating the importance of McArdle’s question posed at the beginning of this article: ‘When thinking about any repeated measures analysis it is best to ask first, what is your model for change?’ (McArdle, 2009, p. 579). Alternative longitudinal SEM approaches that address specific questions with differing strengths and weaknesses include the autoregressive latent trajectory (ALT) model (Bollen and Curran, 2004), survival models (Newsom, 2015, chapter 12), continuous time models (Driver et al., 2016), simplex models (Newsom, 2015), the incorporation of definition variables (Mehta and West, 2000), regime switching LCS models (Chow et al., 2013), latent trait-state models (Steyer et al., 1999), and extensions of latent curve models including structured residuals and time-varying covariates (Curran et al., 2014). For a general introduction to longitudinal SEM approaches we recommend the recent book by Newsom (2015).

Direct comparisons of LCS models to competing models exist but are relatively rare. Usami et al. (2016) compared the LCS to the auto-regressive cross-lagged factor (ARCL) model, and showed lower levels of bias in the parameter estimates of the LCS model, depending on the number of time points and sample size, but slightly more power for the ARCL model (due to decreased standard errors). Notably, they observed that model selection was best when using the less conventional approach of comparison model fit indices (RMSEA and CFI) as opposed to likelihood ratio tests or information indices. Using simulation studies, McArdle and Hamagami (2001a) compared the bivariate dual change score model to a Multilevel Change Score Regression Model under a range of known data generating processes. They showed that the multilevel regression model performed adequately only under a range of restrictive conditions including no missing data, an absence of error terms and no bivariate coupling. The bivariate dual change score model on the other hand was able to accurately recover parameter estimates under a range of missingness conditions, even up to the extreme case of cross-sectional data (i.e. one timepoint per individual), as long as data was Missing Completely at Random (p. 233), illustrating the robustness and flexibility of LCS models. Compared to simpler, more traditional techniques LCS and related models more natural accommodate a range of commonly encountered research challenges, including missing data, unequal spacing, time-varying covariates, and latent and manifest group comparisons which may aid in the nature, direction and precision of statistical inferences in studying dynamic processes (Curran et al., 2010). Many developmental hypotheses can be cast as a special case of the LCS, but researchers should always carefully consider whether a given model best captures their central developmental hypotheses compared to other analytical approaches.

## 5 Fitting latent change score models using open source software

A wide array of tools exist to fit longitudinal SEM models, ranging from modules within popular statistical tools (e.g., AMOS within SPSS; Arbuckle, 2010) to dedicated SEM software (e.g., Mplus; Muthén and Muthén, 2005). We focus on two freely available tools: The package lavaan (Rosseel, 2012) within R and a standalone, GUI-based tool Ωnyx (von Oertzen et al., 2015).

### 5.1 Lavaan

R (R Development Core Team, 2016) is a powerful programming language with a rapidly growing user community dedicated to data analysis and visualisation. Several excellent interactive introductions to R exist, including http://tryr.codeschool.com/ or http://swirlstats.com/. The core strength of R is the wide range of packages dedicated to addressing specific challenges (more than 10,000 as of February 2017), implementing statistical techniques, visualisation and more. Several packages dedicated to SEM exist, including OpenMx (Boker et al., 2011) which allows for a high degree of model specification flexibility, but relatively complex syntax, the sem package (Fox, 2006), Bayesian SEM (blavaan, Merkle and Rosseel, 2015), regularized SEM for complex models (regsem, Jacobucci et al., 2016) and even a new package dedicated specifically to specific subtypes of longitudinal SEM (RAMpath, Zhang et al., 2015).

We will focus on lavaan (Rosseel, 2012) as this is a highly popular and versatile tool for modelling various structural equation models, including longitudinal models. Lavaan syntax consists of multiple lines specifying relations among variables using different operators for e.g. factor loadings (‘=∼’), regressions (‘∼’), (co)variances (‘∼∼’), and means or intercepts (‘∼1′). In the syntax below we specify a simple, univariate latent change score model, estimating five key parameters (in bold).

**Table:** 

#Fit the Univariate Latent Change Score model in Lavaan to simulated data	
LCS<-'	# Specify the model name
COG2 ∼ 1*COG1	# This parameter regresses COG2 perfectly on COG1
dCOG1 =∼ 1*COG2	# This defines the latent change score factor as measured perfectly by COG2
**dCOG1 ∼1**	**# This estimates the conditional mean of the change score**
**COG1 **∼** 1**	**# This estimates the mean of COG1**
COG2 ∼ θ*1	# This constrains the intercept of COG2 to θ
**dCOG1 ∼∼ dCOG1**	**# This estimates the conditional variance of the change scores**
**COG1 ∼∼ COG1**	**# This estimates the variance of the COG1**
COG2 ∼∼ θ*COG2	# This fixes the variance of the COG2 to θ
**dCOG1∼COG1**	**# This estimates the self-feedback parameter**
fitLCS <- lavaan(LCS, data = simdatLCS,estimator = 'mlr',fixed.x = FALSE,missing = 'fiml')	
## this fits the model	
summary(fitLCS, fit.measures = TRUE, standardized = TRUE, rsquare = TRUE)	
## this reports model fit	

Lavaan example syntax. Comments in R are preceded by #. Key LCS parameters are boldfaced.

The lavaan syntax and simulated data for all five model types discussed above is available online https://osf.io/4bpmq/files/. These scripts install and load the relevant packages if needed, simulate data according to given set of parameter values, visualize raw data and fit the model. For a simulated data object called ‘simdatLCS’, the syntax above fits a simple Univariate Latent Change Score model with a Yuan-Bentler correction for non-normality (‘estimator=’mlr’), and full information maximum likelihood to deal with missing data (‘missing=’fiml’). In Appendix A we provide a step-by-step instruction to fit models using R or Ωnyx (see below).

### 5.2 Ωnyx

Although syntax-centred methods for SEM are most common, new users may prefer a more visual, path model based approach (e.g. AMOS, Arbuckle, 2010). One powerful tool is Ωnyx (von Oertzen et al., 2015), a freely available graphical modelling software for creating and estimating various structural equation models. At the time of writing, we used the most recent public version (Ωnyx 1.0-972), available from http://onyx.brandmaier.de/. Ωnyx provides a purely graphical modelling environment without a model syntax level, that is, models are simply drawn as path diagrams. As soon as datasets are loaded within a model, estimation starts on-the-fly and parameter estimates will be directly shown in the model diagram. In addition to its easy-to-use interface, a particular strength of Ωnyx is its capability of generating model syntax for other programs, such as Lavaan (Rosseel, 2012) OpenMx (Boker et al., 2011), or Mplus Muthén and Muthén, 2005). The focus on the graphical interface makes Ωnyx especially useful for beginners who want to get a basic comprehension of SEM, but also for more advanced users who either want to transition to other SEM programs or need to produce diagrams for presentations or manuscripts. Finally, Ωnyx provides template models for commonly used models, reducing time needed to set up standard models to a minimum. Here, we will give a brief introduction on how the Ωnyx graphical user interface works.

**Fig. 6:**
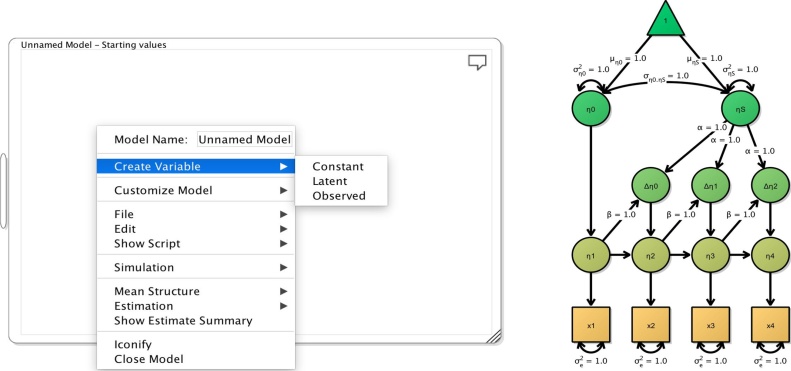
Ωnyx interface.

The idea behind Ωnyx is a little different to typical editors. The main menu is virtually empty (with the exception of basic load and save functions) and there is neither a tool ribbon (e.g., as in Microsoft Word) or a toolbox (e.g., as in Adobe Photoshop) to access functions. Instead, Ωnyx relies heavily on context-menus that are accessible with right mouse-clicks. A double-click performs a context-specific default action. For example, when Ωnyx is started, the empty Ωnyx desktop is shown. A right-click on the desktop opens a new model frame, which is a container for a SEM. Alternatively, a double-click on the desktop creates a model frame (see Fig. 6 for an example of the interface). In Appendix A, we provide a step by step guide to fitting an existing model to data within Ωnyx, as well as a step-by-step explanation how to specify a new model from scratch.

### 5.3 Developing intuitions about change using an interactive shiny app

**Fig. 7:**
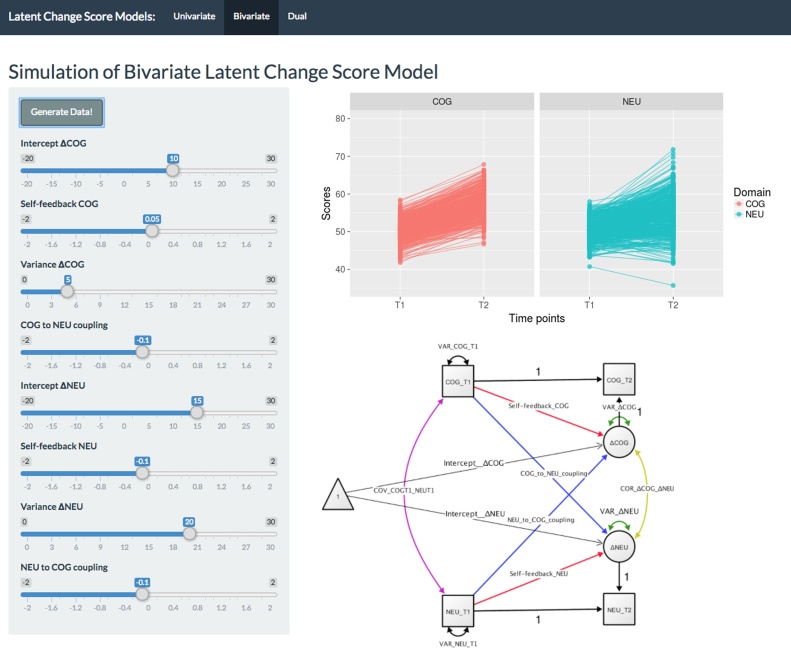
Shiny interface. At the top users can select from three different latent change score models (Univariate, bivariate or dual). At the left, users can modify key parameters and select ‘generate data’ to simulate data with a given parametrisation. On the right, the raw data as well as the path model will shown. This allows users to form an intuition of the effect of dynamic coupling. For instance, it can illustrate that even in the absence of significant changes within a domain, a coupling parameter from another domain can cause significant increases or decreases over time.

Above we explained the basics of LCS models, including graphical representations. Although these examples are relatively easy to understand, one challenge with complex dynamic models is that it can be hard to intuit what the consequences of changes in various parameters might be. To ameliorate this problem, we have built an interactive online tool using the R package Shiny (Chang et al., 2016). This tool allows researchers to modify the key parameters of interest for three key models (univariate latent change score, bivariate latent change score, and bivariate dual change score) in an interactive fashion and examine the consequences for the observed scores. Fig. 7 illustrates our shiny interface, which can be found at http://brandmaier.de/shiny/sample-apps/SimLCS_app/.^4^ The drop-down menu at the top can be used to select one of three models, and the sliders can be used to tweak individual parameters. Changing the key parameters causes the underlying simulation to be modified on the fly, and the panels at the bottom visualize the raw data simulated. The underlying code can easily be accessed and modified, such that researchers can tailor our code to their specific research design. Our hope is that this tool will prove useful in developing intuitions about dynamic co-occurring processes of development and change.

## 6 Examples

Below we illustrate the flexibility of Latent Change Score modelling by describing two empirical examples. First, we describe cognitive (processing speed) and neural (white matter fractional anisotropy) changes from a training intervention study in younger and older adults. Second, we describe group differences in structural changes (i.e. cortical thinning) in a developmental study of (late) adolescents aged 14–24. These applications illustrate the types of questions naturally accommodated by latent change score models.

### 6.1 Correlated change in high intensity training intervention: the COGITO sample

The first illustration comes from data on the COGITO project (Schmiedek et al., 2014), a high-intensity (100 day) training intervention with pre and post-tests cognitive scores for 204 adults: 101 young (age M = 25.11, SD = 2.7, range = 20–31) and 103 old (age M = 70.78, SD = 4.15, range = 64–80).

We examine changes between pre- and post-test scores on a latent variable of processing speed, measured by three standardized tests from the Berlin Intelligence Structure test measured on two occasions (see Schmiedek et al. (2010) for more details). The neural measure of interest is fractional anisotropy in the sensory subregion of the Corpus Callosum (see Lövdén et al., 2010 for more details – note in our exploratory analysis this subregion gave the most stable results and so it was used for our illustration). Longitudinal neuroimaging data was available for a subset of 32 people (20 younger, 12 older adults). We fit the model to the entire sample using Full Information Maximum Likelihood estimation, maximizing the use of our sample and decreasing bias compared to complete case analysis. However, the neural parameters should be interpreted with a level of caution commensurate to the modest sample size. See Lövdén et al. (2014) for further discussion regarding the benefits of FIML in such a context and Enders (2001) for more general discussion of FIML.

**Fig. 8:**
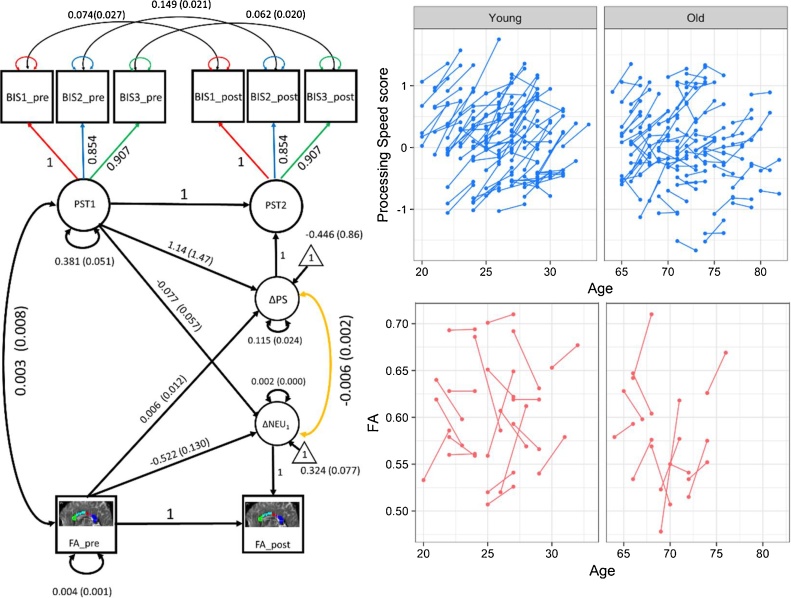
COGITO correlated change in processing speed and white matter plasticity. The panels on the left show the fitted model, parameter estimates and standard errors. The latent variable Processing Speed is measured by three subtests of the Berlin Intelligence Structure test (BIS1-BIS3) measured before (pre) and after (post) an intensive training intervention (see Schmiedek et al., 2010). Observed variable means and variance estimates are omitted for visual clarity. The panels on the right show the raw scores changing across two occasions. The raw scores are plotted on separate panels to accommodate the age gap, but the model is estimated for the population as a whole.

First, we test a multiple indicator univariate latent change score model (the same type of model as shown in Fig. 3). This univariate (only processing speed) multiple indicator (a latent variable of processing speed is specified) latent change score model fits the data well: *χ*^2^(12) = 15.052, *P* = 0.239, RMSEA = 0.035 [0.000 0.084], CFI = 0.996, SRMR=0.028, Yuan-Bentler scaling factor = 0.969. Inspection of key parameters shows that scores increased between pre- and post-test (the intercept of the change factor = 0.224, se = 0.031, Z = 7.24), there were significant individual differences in gains (variance parameter for the latent change score: est = 0.120, se = 0.019, Z = 6.5, but the rate of improvement did not depend on the starting point: est = −0.069, se = 0.054, Z = −1.32). Next, we include a neural measure, namely Fractional Anisotropy in the sensory region of the Corpus Callosum measured pre- and post-test, to fit a *bivariate* (neural and behaviour) *multiple indicator* (we include a measurement model) *latent change score model* shown in Fig. 8.

We next test the evidence for four possible brain-behaviour relationships: *Covariance* (are scores on processing speed at T1 *correlated with* white matter structure at T1?), neural measures as leading variable (do differences in white matter integrity at T1 affect the rate of cognitive training gains?), cognition as leading variable (do processing speed scores at T1 predict degree of white matter plasticity between T1 and T2?) and/or *correlated change* (is the degree of improvement in the cognitive domain correlated with the degree of white matter change in individuals?). Fig. 8 shows the full model, as well as the changes in processing speed factor scores (top right) and fractional anisotropy (bottom right) (note we artificially expanded the interval between testing intervals for visual clarity). First, we find that model fit is good: *χ*^2^(20) = 24.773, *P* = 0.21, RMSEA = 0.034 [90% CI: 0.000 0.072], CFI = 0.995, SRMR = 0.083, Yuan-Bentler scaling factor = 1.021. The full model is shown in Fig. 8. Inspection of the four parameters of interest, reflecting the four possible brain-behaviour relationships outlined above, shows evidence (only) for *correlated change*. In other words, those with greater gains in processing speed were, on average, those with *less* positive change in fractional anisotropy after taking into account the other dynamic parameters (est = −0.006, se = 0.002, z = −2.631). Although counterintuitive, a similar pattern was also observed in Bender et al. (2015) who observed negative correlation between age-related declines in episodic memory and white matter integrity, such that a greater decrease in fractional anisotropy was associated with greater improvement in episodic memory (whereas at T1 FA and episodic memory were positively correlated). This illustration shows how LCS models can be used to simultaneously estimate four rather distinct brain-behaviour relationships over time.

### 6.2 Multigroup analysis of prefrontal structural change in late adolescence: the NSPN cohort

As the example in the Cogito sample shows, LCS offers a simple and powerful way to test distinct dynamic pathways within a single LCS model. However, investigations in Developmental Cognitive Neuroscience are often concerned with differences between groups (e.g. gender, treatment vs. controls, psychopathology vs. healthy controls, low vs high SES etc.). Such questions are best addressed by means of *multigroup modelling*. Here we illustrate a multigroup model to compare structural changes in a group of adolescents. Data for this is drawn from the Neuroscience in Psychiatry Network (NSPN), a cohort that studies development in adolescents (see also Kiddle et al., 2017, Kievit et al., 2017, Whitaker et al., 2016) Here we illustrate a multigroup model to compare structural brain change in a group of adolescents. Previous work suggests differences in the temporal development of the frontal cortex, with boys generally maturing later than girls (Giedd et al., 2012, Ziegler et al., 2017), possibly as a consequence of differences in sensitivity to hormone levels (Bramen et al., 2012).

For our analysis, we focus on volumetric changes in the frontal pole. This region is part of the frontal lobe, which is often discussed with respect to the speed of maturational changes and its purported role in controlling higher cognitive functions and risk taking behaviour (e.g. Crone and Dahl, 2012, Johnson, 2011, Mills et al., 2014).

**Fig. 9:**
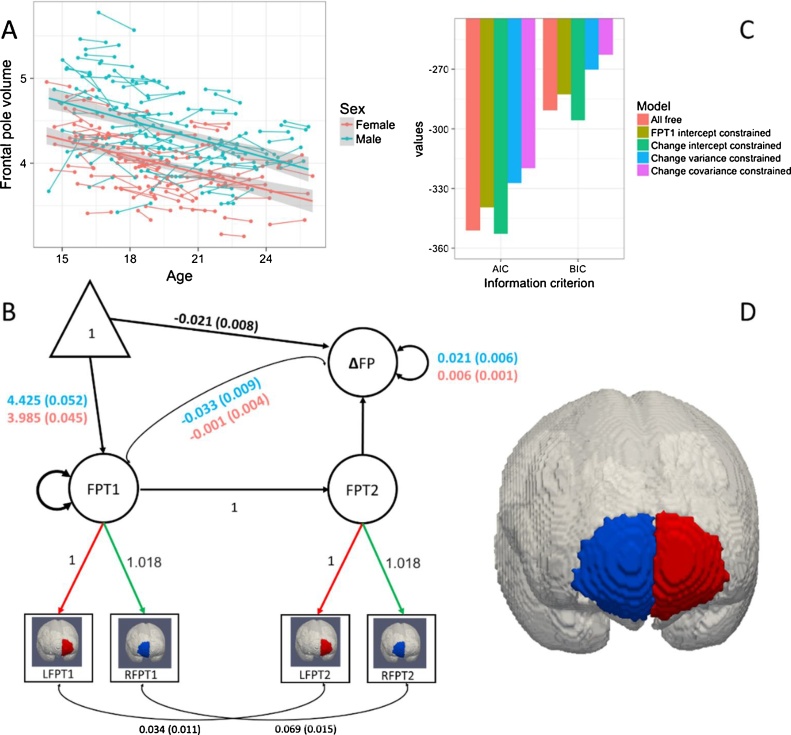
NSPN: Differential variability in frontal lobe thinning. Panel A shows longitudinal development in frontal structure. Panel B shows the model fit for the best model. Where parameters are different between groups we show male estimates in blue (top parameter), female in red (bottom parameter). Panel C shows the AIC and BIC of the free versus constrained models – in all cases only one parameter is constrained to equality and compared to the ‘all free’ model. Panel D shows the left and right frontal poles of the neuromorphometrics atlas used in our analysis. See Appendix B for more details on the imaging pipeline. (For interpretation of the references to colour in this figure legend, the reader is referred to the web version of this article.)

Our sample consisted of 176 individuals, mean age = 18.84, range 14.3–24.9, 82 girls, scanned on two occasions (average interval: M = 1.24 years, SD = 0.33 years). We fit a multiple indicator univariate latent change score model, with volume of the frontal pole (FP) using the neuromorphometrics atlas as the key variable (see Fig. 9D for an illustration). Our measurement model consisted of volumetric estimates of the left and right FP measured on two occasions (for more details on the structural processing pipeline, see Appendix B). We can use the framework of multigroup models to investigate whether there is evidence for differences between the two groups in the key parameters of interest. The four parameters of interest are the mean of the frontal factor (reflecting mean volumes at T1), the intercept of the change factor (reflecting the rate of change), the variance of the latent change scores (reflecting individual differences in rates of FP change) and the covariance between FP at T1 and rate of FP change. To do so, we employ the method of equality constrained Likelihood Ratio tests, by comparing a model where some parameter of interest is constrained to be the same across the two groups with a model where the parameter is allowed to be free. The difference in model fit under the null hypothesis is chi-square distributed with a df equivalent to the difference in numbers of parameters being constrained. In other words, if a parameter of interest is the same (or highly similar) between two groups, the chi-square test will fail to be rejected, suggesting the more parsimonious model is sufficient.

First, we fit a model where all measurement model parameters (constraining all factor loadings and residual (co)variances) are constrained to be equal across males and females, but all other parameters are free to vary between the sexes. This model fit the data well: *χ*^2^(9) = 8.929, *P* = 0.44, RMSEA = 0.00 [0.000 0.120], CFI = 1, SRMR = 0.021, Yuan-Bentler scaling factor = 0.983. Next, we explored which (if any) of the four parameters above differed between the sexes. If a parameter is different between the groups, constraining it to be equal should result in a significant decrease in model fit. Using the likelihood ratio test, we observe significant decreases in model fit by constraining the mean of frontal lobe volume at T1 to be equal across the sexes (χ2Δ = 38.01, dfΔ = 2, p = <0.0001). Inspection of parameter estimates shows, unsurprisingly, greater FP volume in males, compatible with either larger brains, delayed cortical thinning, or a combination of the two. Contrary to expectations, constraining the intercept of the change scores did not lead to a significant decrease in fit (χ2Δ = 0.31889, dfΔ = 2, p = 0.57), indicating an absence of reliable differences in cortical thinning. However, constraining the *variance* of change scores to be equal did result in a significant drop in fit (χ2Δ = 49.319, dfΔ = 2, p = <0.0001), with males showing greater individual differences in rates of thinning than females see also Ritchie et al., (2017). Finally, constraining the covariance between frontal volume and change scores also led to a drop in model fit, with males showing a stronger (negative) association between volume at T1 and rate of change (compatible with the hypotheses of delayed development in males). Fig. 9 shows the temporal development of FP structure between sexes, information based model comparison and parameter estimates for the full model (with different estimates for males and females where required).

Together, this suggests that there are considerable differences in frontal development between males and females in (late) adolescence: Although males and females show similar rates of cortical thinning, males show greater initial volume, greater individual differences in thinning and a stronger association between initial volume and rate of thinning. Notably, the parameters where the evidence for sex differences is strongest (e.g. variance and covariance in change scores) are not parameters often studied using conventional techniques such as paired *t*-tests (other than as a statistical assumption such as equality of variances). Conversely, the parameter that would be the key focus with traditional techniques (i.e. group differences in change scores) does not show differences.

## 7 Conclusion

In this tutorial, we introduce the powerful framework of Latent Change Score modelling whose deployment can be an invaluable aid for developmental cognitive neuroscience. It is our hope that more widespread employment of these powerful techniques will aid the developmental cognitive neuroscientific community. Adopting the statistical techniques we outline in tandem with the more widespread availability of large, longitudinal, cohorts of developing adolescents will allow researchers to more fully address questions of interest, as well as inspire new questions and approaches. The approach we outline puts renewed emphasis on the value of longitudinal over cross-sectional data in addressing developmental questions.

## Conflict of interest

E.T.B. is employed half-time by the University of Cambridge and half-time by GlaxoSmithKline; he holds stock in GlaxoSmithKline.

## Funding

RAK is supported by the Sir Henry Wellcome Trust (grant number 107392/Z/15/Z) and the UK Medical Research Council Programme (MC-A060-5PR61). The NSPN cohort was supported by a strategic award by the Wellcome Trust to the University of Cambridge and University College London (095844/Z/11/Z). This project has received funding from the European Union’s Horizon 2020 research and innovation programme under grant agreement No 732592.

## Figures and Tables

**Figure:**
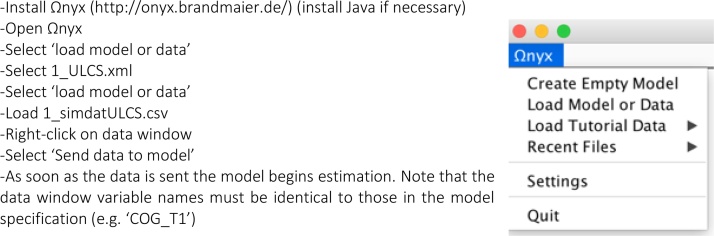


**Figure:**
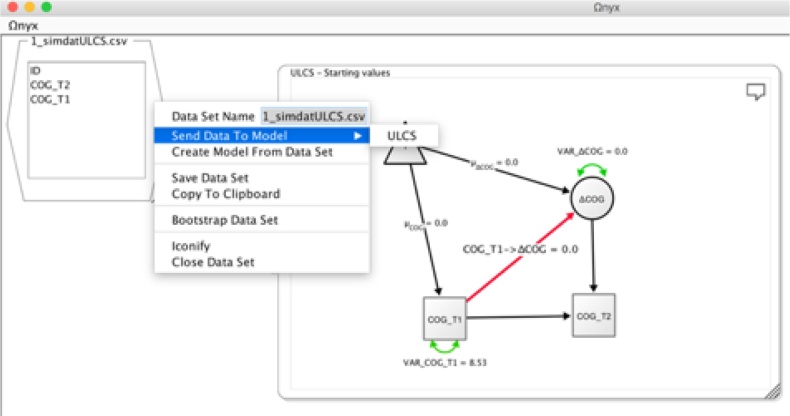


## Appendix A

**Creating and fitting Latent Change Score models using R and Ωnyx**

In the associated folder you will find code (file extensions .R), data (file extensions .csv) and Ωnyx model files (file extensions .xml) for five different latent change score models. Below we outline how to specify and fit models using lavaan and R.

1 Analyse data using R and lavaan

–Install R (https://cran.r-project.org/)

–Install Rstudio (recommended) (https://www.rstudio.com/)

–Open the relevant lavaan file (e.g. ‘1_ULCS.R’)

–Install the appropriate packages by uncommenting (e.g. lines 17–19 in 1_ULCS.R)

–Select and run lines 30–60 to create a simulated dataset with given parameters

–Select and run lines 85–92 to visualize the raw data

–Select and run lines 65–80 to fit the latent change score model

–Run line 81 to examine model fit and parameters

–If so desired, modify the parameters in lines 47–53 to examine the consequences for the raw data and model fit

2 Fit a model to existing dataset using Ωnyx

3 Creating a new model using Ωnyx

To create variables in the model, right-click on the model frame and choose ‘add variable’ to add an observed, latent or constant variable. Alternatively, users can double-click on the model frame to create an observed variable. Existing variables can be moved by left-dragging (press left mouse button and move mouse while button pressed). Double-clicking while holding down the SHIFT button creates a latent variable. Regression paths (single headed) are drawn by right drags, that is, by pressing the right mouse button on a variable and releasing the button only when the mouse was moved to a second variable. Covariance paths (double headed) are drawn by holding SHIFT while releasing a path. Variance paths are created by creating a covariance path from a variable to itself. By default, path values are fixed to one. Paths values can be changed either by right-clicking a path and entering a new value in the context menu or by moving the mouse over a path and directly typing the desired value. Path can be defined to represent a freely estimated parameter by right-clicking a path and choosing “Free Parameter”. Using the context menu, parameters can be renamed and starting values can be given. Observed variables can be either associated with a data column or not. This is indicated by observed variables either having a grey box (not linked) or a black box (linked data). To link a variable with data, one can load a dataset from an existing file and simply drag the variables onto the Ωnyx representation.

As an alternative to the manual creation of a change score model, Ωnyx provides a wizard for quick model specification, even for more advanced models. Users can right-click on the Ωnyx desktop and choose, among other models, ‘Create new LGCM’ (for a linear growth curve model) or ‘Create new DCSM’ (for a dual change score model), which can then be modified as desired. The LCSM wizard allows you to specify the number of time points. Once the model is specified, users can simply drag and drop data (e.g. columns in a .csv file) from an existing data file onto the appropriate observed variables in the model by selecting ‘load data’ from the main drop-down menu. As soon as variables are added the program starts estimation.

*Advanced features of Ωnyx*

As noted before, Ωnyx provides various functions to export models to other SEM programs. For example, if users wish to use more complicated modelling approaches (e.g., ordinal outcomes, non-linear constraints), Ωnyx can quickly create a graphical model specification which then can be exported to another SEM program that allows greater flexibility in modelling. Ωnyx also exports diagrams as bitmap graphics (JPG, PNG) or vector graphics (PDF, EPS) and even creates LaTeX-representations of diagrams based on the tikz package. Ωnyx comes with different visual style templates that can be applied to a model with a single click. To choose among styles, right-click on a model frame and choose Customize Model → Apply Diagram Style, or hit CTRL+L to cycle through styles in the active model frame. Ωnyx may be used to simulate data from the model-implied means and covariance matrix. To this end, choose “Simulation-> Start Simulation” in the context menu of a model frame. Ωnyx allows parameter names to be defined in a pseudo LaTeX input style, which allows users to use greek symbols (e.g., alpha, beta, gamma,…), subscripts (e.g., epsilon_i) and superscripts (e.g., sigma^2).

## Appendix B

**Structural Imaging and Map Generation**

Brain scans were acquired using the MPM protocol (Weiskopf et al., 2013) on three 3T whole-body MRI systems (Magnetom TIM Trio; VB17 software version; Siemens Healthcare) located in Cambridge and London. Between-site reliability of MRI procedures was assessed in a pilot study scanning five healthy volunteers at each site. The between-site bias was found to be less than 3% and the between-site coefficient of variation was less than 8% for both longitudinal relaxation rate (R1) and MT parameters (Weiskopf et al., 2013) Isotropic 1 mm MT images were quantified in Matlab (2014b, The MathWorks, Inc.) using SPM12 r6685 (Wellcome Trust Centre for Neuroimaging, London, UK, http://www.fil.ion.ucl.ac.uk/spm), the Voxel-Based Quantification (VBQ) toolbox for SPM (Callaghan et al., 2014) and custom made tools.

**Longitudinal Image Processing and Feature Extraction**

All further image processing steps of MT maps were performed in SPM12. Longitudinal morphometry was used to assess macroscopic brain maturational changes. Since longitudinal imaging is prone to artefacts due to registration inconsistency, scanner inconsistencies and age-related deformations of the brains, it requires sophisticated processing pipelines in order to detect the changes of interest and achieve unbiased results.

First, we applied symmetric diffeomorphic registration for longitudinal MRI (Ashburner and Ridgway, 2012) combining non-linear diffeomorphic and rigid-body registration and correction for intensity inhomogeneity artefacts. The optimization is realized within one integrated generative model and provides consistent estimates of within-subject brain deformations over the study period. The registration model also creates a midpoint image for each subject and the corresponding deformation fields for every individual scan.

Second, we applied SPM12’s Computational Anatomy Toolbox (CAT, r955, Structural Imaging Group, http://dbm.neuro.uni-jena.de/cat12/) segmentation to each subject's midpoint image, which assumes every voxel to be drawn from an unknown mixture of gray matter (GM), white matter (WM), cerebrospinal fluid (CSF). Earlier results showed that MT maps are highly suitable for automated segmentation in multi-subject morphometric studies, showing improved GM tissue contrast in subcortical structures (Helms et al., 2009). This applied segmentation procedure contains partial volume estimation (PVE) to account for mixed voxels with two tissue types (Tohka et al., 2004). The CAT algorithm is based on an adaptive maximum a posteriori (AMAP) approach (Rajapakse et al., 1997) and subsequent application of a hidden Markov random field model (Cuadra et al., 2005). Importantly, the applied AMAP estimation does not rely on tissue priors, which overcomes potential bias due to the application of inappropriate tissue priors in young maturing subjects with different to adult brain anatomy.

Third, nonlinear template generation and image registration was performed on the individual midpoint GM and WM tissue maps using DARTEL registration and the template was registered to MNI space using an affine transform (Ashburner, 2007). Consecutively longitudinal normalised tissue segments from all subjects and time-points were modulated by Jacobian determinants accounting for local tissue volume differences across subjects and within-subject changes over time. In order to detect stronger deviations due to potential segmentation or normalization errors, we included a quality check using covariance-based sample inhomogeneity measures implemented in the CAT toolbox to exclude subjects with extremal values and/or severe artefacts.

Fourth, neuromorphometrics atlas was used to assess gray matter density in the bilateral frontal poles after Gaussian smoothing with 6 mm full width at half maximum. The atlas was based on maximum probability tissue labels derived in the MICCAI 2012 Grand Challenge and Workshop on Multi-Atlas Labeling with data originating from the OASIS project (http://www.oasis-brains.org/) and the atlas provided by Neuromorphometrics, Inc. (http://Neuromorphometrics.com/) under academic subscription.

All longitudinal features for subsequent structural equation modelling were obtained using the above steps. Since common processing pipelines of longitudinal and cross-sectional data is different, this can introduce biases (Bernal-Rusiel et al., 2013). We initially focussed on the largest fully longitudinally processed subsample of the NSPN dataset available, with 202 subjects having at least 2 scans per person. After rigorous quality control of all processed imaging data, scans from 26 subjects had to be discarded due to artefacts, resulting in a finally analyzed brain features from 373 scans from 176 subjects (2.12 scans/person). Although a small subset of individuals had a third intermediate scan, for modelling purposes here we use only the first and last scan.

## Footnotes

1 It is worth noting that such hypotheses of temporal precedence in measurable properties do not imply a dualist perspective on mental and physical processes (cf. Kievit et al., 2011). They do suggest scientifically relevant distinctions can be made with implications for interpretation, the likely consequences of interventions and early detection of non-typical development.

2 Although it should be noted that SEM software packages can vary in their default model specifications, so users should always be aware of these modelling assumptions

3 https://osf.io/4bpmq/?view_.

4 Note: some firewalls block the app. A zipped folder that contains all scripts and can be run locally is available on https://osf.io/4bpmq/files/.
